# Some Injuries of the Eyeball*Read before Medical Association of Georgia, April, 1896.

**Published:** 1896-08

**Authors:** S. Latimer Phillips

**Affiliations:** Savannah, Ga.


### SOME INJURIES OF THE EYEBALL.*

BY S. LATIMER PHILLIPS, M. D., SAVANNAH.

Among the emergency cases met with in daily practice, injuries of the eyeball play a more or less important part. We have them from a mere scratch of the cornea or conjunctiva to complete evisceration of the contents of the eyeball. Without further preamble I shall report a few cases that have come under my observation:

CASE I.—*Blow on Eye; Rupture of Choroid and Hemorrhage into Vitreous; Partial Recovery of Sight.*—Maggie T., æt. 10, came to consult me with the history of having been struck in the left eye, one week before, while chopping wood, a piece of the axe-helve splitting away and striking eye. Lids were swollen and eye painful. On inspection of the eye, found, to nasal side, the conjunctiva torn up from the sclerotic where the foreign body struck the eye, but no foreign matter was visible, and there was no apparent puncture of the sclera. Iris was torn away from its periphery at upper centre, perhaps for a fourth of its extent; pupil dilated, but with opthalmoscope could get no reflex from fundus. There was no light appreciation, Lx. I then had the eye bandaged and had patient put upon gr. ⅛ muriate pilocarpine every night. Six days later on, eye painless, light perception and I could get red reflex from fundus. Now, with the opthalmoscope I found large quantities of floating bodies in vitreous, so much so, that details of the fundus could not be made out. Twenty-three days from time I first saw patient vision had been brought up to $\frac{10}{c}$ from nothing, the result of the clearing of the vitreous. Eye had now become quiet and tension normal. There had been no injury of the lens, but I found a large rent in the choroid near the centre. I saw the patient at intervals for several years, but vision remained the same. The vitreous cleared up, but the large spot of choroidal atrophy still remains.

---

*Read before Medical Association of Georgia, April, 1896.

In this case the foreign body struck the eye with a great deal of force, but in such a position that the sclerotic resisted, being tough and elastic. The more tender choroidal vessels gave way, and there was a profuse hemorrhage into the vitreous chamber, thus producing over-tension and pain. The pilocarpine acted well in this case, lowering tension and bringing about the absorption of the blood in the vitreous.

Case II.—*Burn of Eye with Hot Iron; Non-penetration of Body; Infection and Ulceration; Recovery.*—Thomas F., *æt.* 55, blacksmith by trade. A few days before I saw him, had been struck in left eye by a piece of hot iron. When he came to me, eye was painful and lids swollen, conjunctiva chemotic and overhanging corneal border. In centre of cornea was the point of injury. The foreign body had struck the eye sufficiently hard to penetrate, or almost penetrate the cornea, and had then rebounded. There was no sign of its having passed through the lens, or having lodged in the vitreous or the coats back of it. In the right eye there was a fully developed cataract, with only light perception; hence it was all the more urgent that sight, if possible, be preserved in the left eye. Eye was thoroughly washed out with a saturated solution of boracic acid, and atropia instilled; but notwithstanding this, the injured part of the cornea ulcerated and in a few days hypopyon was observed. Three times was paracentesis of the anterior chamber done to evacuate the pus, and after six weeks the ulceration healed, leaving a small central scar. The sight was fair, enabling him to return to his work of making a living with comfort.

In this case there was evidently a previous conjunctival catarrh. The iron striking the cornea, rebounded, but so bruising the corneal tissue that the germ of catarrh found ready access to the wound, thus infecting it.

Case III.—*Injury to the Cornea, Iris, and Lens from Explosion Breaking Glass Bottle, the Latter Cutting Eye; Traumatic Cataract.*—Walter M., *æt.* 8, was brought to me from the country by his father for an injury of the left eye, received four days previous. He had put some gunpowder in a bottle and dropped a lighted match in to see what the result would be. He found out very soon by a piece of the flying glass striking him in the eye. It hit the cornea to inner side of centre, making three diverging cuts—one towards centre, one up, and one in. Iris cut clear to its base along horizontal median line, pupil semi-dilated and drawn toward the iris fissure, very little irritation about the eye now; no pain, tension normal, lens opaque, evidence of having been hemorrhage into anterior chamber from cut surface of iris, but now absorbed; has only light perception. Eye was treated with atropia locally and bandaged. When pupil dilated found two adhesions of iris to lens capsule. At end of two weeks, all irritation having passed away, I made a small opening in the lens capsule hoping to hasten absorption, and not knowing when I should ever see the patient again, if at all. And as I expected, I have never seen or heard from him since. My presumption was there was no foreign body in the eye, from the fact that the eye soon lost all signs of irritation and there was no pain or discomfort on pressure of the eyeball. If the one needling was not sufficient to produce absorption of the lens, if he comes back, another can be done easily, with fair prospects of good sight.

CASE IV.—*Struck on Eye by Cork from Exploding Soda-water Bottle; Hemorrhage in Vitreous.*—J. H. M., *æt.* 20, of Jesup, Ga. While opening a soda-water bottle two days since, there was an explosion, cork striking him with considerable force in left eye. Immediately he noticed he was blind in that eye. When I saw him I noted the following conditions: Bruised condition of lids and a painful eye, with tension above normal; only light appreciation, pupil semi-dilated; no hemorrhage in anterior chamber, but no reflex could be gotten of fundus from hemorrhage in vitreous, cornea hazy. Iris was torn toward nasal and toward temporal side, the split occurring near pupil and running toward ciliary border above. As he had to return at once to his home, I saw him no more. He had atropia locally and was to have taken gr. $\frac{1}{6}$ pilocarpine muriate that night.

In this case there was rupture of iris and probably choroid. After the absorption of the blood in the vitreous chamber, the eye possibly became good, much depending though, upon the amount of injury to the choroid.

CASE V.—*A Unique Method of Injury Involving Cornea, Iris, and Lens.*—L. C., 7 years of age, while playing in street two days before I saw him, was struck in the eye by one of his playfellows with a most peculiar arm of warfare. It seemed that he had gone out with a cake or something in his hand which his playmates begged him for, but declining, they became incensed and began an attack upon him. One of them picked up a bird's head that he found on the ground and threw it at the child, striking him in the eye with the sharp beak, cutting cornea and iris, and penetrating lens. I found the cornea cut from the centre vertically to near ciliary region. Through the wound in the cornea the iris was to some extent punctured, displacing the pupil. There was present a traumatic cataract, also redness and pain in eye. He could only notice shadow of hand when passed in front of eye. After the use of cocaine, whatever iris tissue protruding through corneal wound that could be caught up with iris forceps was cut off, atropia dropped in eye and bandage applied. Directions were given that cold applications be used on eye at intervals, and that one drop of atropia sulph. (gr. iv. to ℥i.) be used in eye every two hours until I saw patient again, which I did in about four hours. There had been no special influence on the pupil by the atropia, but face was flushed and breathing rapid, with skin hot and dry to touch; complains of no dryness of throat. No more atropia used that day, but the next sparingly. Under this treatment the eye soon recovered. The traumatic cataract can be removed any time when he wants it done, with good chances of the restoration of vision.

